# The Role of Multi-Staged Urethroplasty in Lichen Sclerosus Penile Urethral Strictures

**DOI:** 10.3390/jcm11236961

**Published:** 2022-11-25

**Authors:** Enzo Palminteri, Andrea Gobbo, Mirko Preto, Paolo Alessio, Daniele Vitelli, Lorenzo Gatti, Nicolò Maria Buffi

**Affiliations:** 1 Center for Urethral and Genitalia Reconstructive Surgery, Humanitas Cellini, Via Benvenuto Cellini 5, 10126 Turin, Italy; 2 Department of Biomedical Sciences, Humanitas University, Via Rita Levi Montalcini 4, 20072 Pieve Emanuele, Italy; 3 Department of Urology and Reconstructive Andrology, Humanitas Gradenigo, Corso Regina Margherita 8, 10153 Turin, Italy; 4 Urology Clinic—A.O.U. “Città della Salute e della Scienza”—Molinette Hospital, University of Turin, Corso Bramante 88, 10134 Turin, Italy; 5 CURE Group, Department of Urology, Hesperia Hospital, Via Arguà 80, 41125 Modena, Italy; 6 Department of Urology, IRCCS Humanitas Research Hospital, Via Manzoni 56, 20089 Rozzano, Italy

**Keywords:** lichen sclerosus, urethroplasty, buccal mucosa, multistage urethroplasty, patient-reported outcomes, penile urethral stricture

## Abstract

Background: One-stage buccal mucosa graft urethroplasty (BMGU) is advised for non-obstructing, simple penile strictures due to lichen sclerosus (LS), while a multistage approach is preferred for most complex cases. Our study aims to evaluate long-term treatment outcomes and patient-reported outcomes (PROs) in patients undergoing multistage BMGU for LS-associated penile strictures. Methods: This is a retrospective analysis of prospectively collected data on multistage penile BMGU from 2001. All patients underwent a 2-stage (2St) or 3-stage (3St) BMGU with the final closure of the urethral plate. PROs were collected from a pre-defined questionnaire. Results: Twenty patients were successfully treated, while five experienced recurrence. If a first-stage Johanson was only performed, a 3St-BMGU was more likely, and higher treatment success was observed. A time course between the first and last stages shorter than 12 m was an independent predictor of treatment failure. Patients reported high overall satisfaction and urinary flow improvement. Sexual life was not significantly affected, while aesthetic appearance was the most affected dimension. Conclusions: Staged approaches have satisfactory treatment success rates, likely depending on the duration from the first to the last stage. PROs do not differ based on the number of stages performed, and overall satisfaction with the procedure is high.

## 1. Introduction

Lichen sclerosus (LS) is a chronic, inflammatory skin disease with a tendency for the anogenital area that may involve the anterior urethra in a distal to proximal fashion up to its entire length [1,2]. In a contemporary series of 1439 patients, Palminteri et al. found LS responsible for 13.4% of anterior strictures, with a mean length of 7.45 cm [3].

The optimal management of LS strictures is a controversial topic for reconstructive urologists. Buccal mucosa grafts (BMGs) are the reconstructive tissue of choice; as LS is a skin disease, the use of genital skin as substitutive material leads to poor results and should be discouraged [4,5].

Evidence is less conclusive regarding the surgical strategy, and management is entrusted to clinical presentation and the surgeon’s preference. It is commonly accepted that if there is a viable urethral plate, usually >6F, a single-stage BMG urethroplasty (BMGU) can be performed, while if not, a staged BMGU (St-BMGU) should be preferred [6,7,8]. Our experience with complex LS strictures led the surgeon to adopt a staged approach to allow eventual revisions. The treatment we propose in long, obliterating strictures, particularly when a history of previous urethroplasties is present, consists of a 2-stage BMGU (2St-BMGU) or 3-stage BMGU (3St-BMGU). Staged approaches offer the unique opportunity to evaluate graft maturation and healing, excluding its involvement with the disease. Aiming to analyze this staged approach to LS anterior strictures, we reviewed our records and hereby reported long-term outcomes and patient-reported outcomes (PROs).

## 2. Materials and Methods

### 2.1. Patients Selection

Patients were extracted from a prospectively compiled online database instituted at the beginning of the first surgeon activity in our center. Only patients that underwent a 2St-BMGU or 3St-BMGU for LS strictures were included. Before surgery, all subjects were evaluated by retrograde and voiding urethrocystography and uroflowmetry. The surgeon followed the patients personally, and those with a successful treatment were asked for PROs. Treatment success was defined as no further treatments for stricture recurrence, including dilatation and intermittent catheterization. The study was conducted in accordance with the Helsinki Declaration of Ethical Principles and Good Clinical Practices.

### 2.2. Patient-Reported Outcome Measures Evaluation

Considering the highly specific setting of this study, which comprises a staged surgical procedure to both the penis and the urethra, available validated questionnaires on each singular anatomical structure involved were not applicable due to the likely unreliability of the scores. Rather, we have developed a non-validated questionnaire for PROs evaluation, as we believe it better fits this unique surgical cohort. Only patients treated successfully were evaluated due to a high likelihood of biased answers in the group that failed. We adopted a pre-defined questionnaire with the following questions:

1. How do you rate your overall micturition quality?
2. How do you rate your sexual life quality?
3. How do you rate the aesthetical results?
4. Are you satisfied with the general surgical results?

For questions 1, 2, and 3, the answers could be: improved, unmodified, or worsened. For question 4, the answer could be yes or no.

### 2.3. Type of Surgery and Technical Considerations

The surgeon decided to follow a multistage approach preoperatively based on the diagnostic exams and clinical status. The single-stage reconstruction was excluded in obstructing or sub-obstructing strictures (urethral plates < 6F) or in case of a severely diseased and scarred urethra; all strictures were longer than 4 cm.

**Figure 1:**
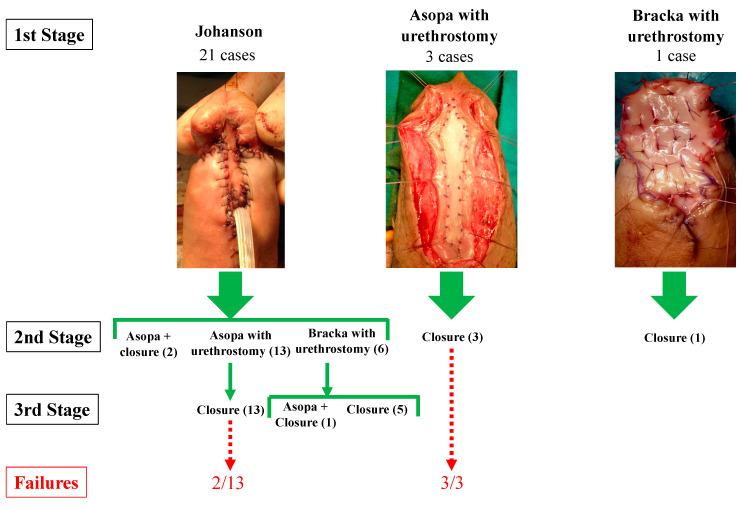
The figure summarizes the surgical management of the patients enrolled in the study with available follow-up. In brackets is reported the number of patients who underwent the procedure, and in red is the number of patients where the treatment failed.

The first stage could consist of 3 different solutions (Figure 1):

(a) A simple opening of the strictured urethra and leaving a penile urethrostomy, as described by Johanson [9];
(b) An opening of the strictured urethra with concomitant dorsal augmentation of the urethral plate with a BMG, as described by Asopa, additionally leaving a penile urethrostomy [10];
(c) An opening of the strictured urethra, complete removal of the diseased urethral plate, reconstruction of an entire new urethral plate with a BMG, as described by Bracka, additionally leaving a penile urethrostomy [11].

A satisfactory intraoperative appearance of the urethral plate at the moment of the second stage prompted the closure of the urethra (with or without BMGU). Conversely, an unviable urethral plate at the second stage resulted in a second “intermediate” stage with BMGU by Asopa or Bracka, leaving an open urethrostomy, with subsequent closure of the urethra at the third and last stage. We define a “viable” urethral plate as one with dry mucosa, of mildly pink color, without extensive scarring or progression of the disease involving the BMG (if BMGU was previously performed). The first surgeon’s evaluation of the criteria was purely subjective based on his experience. The time course between the stages was at least 6 m.

### 2.4. Postoperative Management

#### 2.4.1. First Stage and Second Stage without Re-Tubularization

At the end of the procedure, a 14–16 ch vesical catheter is positioned, and it is removed after 7 days. Both grafted and native exposed urethral plates, immediately after surgery, are medicated with Vaseline gauze and a slightly compressive dressing with double-sided adhesive gauze. The dressing is left untouched for 7 days, and ice is regularly used. Afterward, the dressing is renewed daily with local surgical disinfectants, vitamin E, and hyaluronic acid creams. Between the second and fourth postoperative day, the patient is discharged with detailed indications about subsequent medications. The surgical disinfectant is ceased when the sutured margins are completely closed (about 7–10 days), while vitamin E and hyaluronic acid creams are continued indefinitely. 

#### 2.4.2. Re-Tubularization Stage

The vesical catheter is removed after 3 weeks with a retrograde and voiding cystography. Immediately after surgery, a slightly compressive dressing with double-sided adhesive gauze is performed. Every 2 days, the dressing is renewed in the same fashion until the catheter is removed. The patient is discharged between the second and fourth postoperative days. The surgical site is disinfected for 7–10 days, while vitamin E and hyaluronic acid creams are used for 30 days.

### 2.5. Statistical Considerations

Contingency tables were used to describe population characteristics. Fisher’s exact test and Mann–Whitney test were used to test categorical and continuous variables, respectively. Univariable (U) and multivariable (M) logistic regression models (LRMs) were fitted to evaluate correlation with stricture recurrence. Statistical significance was set at *p* < 0.05. All analyses were performed using STATA^®^17 (StataCorp, College Station, TX, USA).

## 3. Results

Thirty-five patients underwent 2St-BMGU or 3St-BMGU between 2001 and 2015. Table 1 summarizes the main characteristics of the 35 patients that underwent a St-BMGU. All patients had strictures longer than 4 cm. All patients underwent at least one previous surgery to the penis or urethra for LS, four had only circumcisions, eight underwent meatoplasty, and six had surgeries for hypospadias repair. Four patients had a cystostomy in place at the time of surgery, and four were performing periodic dilatations. Seventeen patients had available Qmax from uroflowmetry, and the mean was 6.6 mL/s. 

Ten patients were lost at follow-up (FU); Table 2 summarizes the main characteristics of the population in the study with available FU. The mean age was 46 y (SD 11.12 y), the median FU was 134 m (IQR 77–154 m, range 34–239 m), 20 patients (80%) were successfully treated, while 5 experienced recurrence. 2St-BMGU was performed in 6 patients with 3 failures, while 3St-BMGU was performed in 19 patients with 2 failures. 

If a first-stage Johanson only was performed, age was higher (49 y vs. 33 y, *p* = 0.002), a 3St-BMGU was more likely (96.1% of 3St-BMGU vs. 33% of 2St-BMGU, *p* = 0.001, Table 1), and higher treatment success was observed (90.5% vs. 25%, *p* = 0.016, Table 2). Postoperative complications occurred in 3 patients, and all were fistulas formation surgically resolved, while 1 patient progressed to penile cancer. A time course between the first and last stage shorter than 12 m was associated with the 2St-BMGU procedure (6/9 vs. 2/26, *p*= 0.001, Table 1), with treatment failure (4/6 vs. 1/19, *p* = 0.005, Table 2) and was also a predictor of treatment failure at ULRM (OR 36, *p* = 0.008, Table 3). MLRM controlled for the number of stages performed and confirmed that the time course was an independent predictor of treatment failure (OR = 27, *p* = 0.031, Table 3).

**Figure 2:**
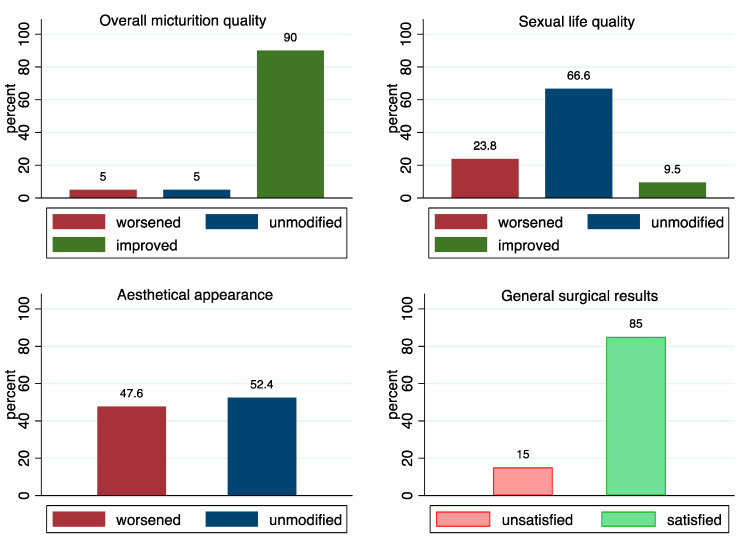
The figure summarizes patients’ answers to the questionnaire. The bars represent the percentage respective to the overall number of answers per question (*n* = 19).

Of the 20 successful treatments asked for PROs, 1 patient was not contactable. Figure 2 summarizes the patients’ answers to the questionnaire. Overall satisfaction was high (17/19), and urinary flow improved in 18/19 patients. Sexual life was not significantly affected, and 14/19 patients reported unchanged, 3/19 worsened, and 2/19 improved function. The aesthetic appearance was unchanged in 11/19 patients and worsened in 8/19. Patients that underwent a final meatus closure not on the top of the glans answered significantly worse in the aesthetic domain (*p* = 0.001, distribution not reported). PROs did not differ based on the number of stages performed.

## 4. Discussion

Urethral reconstruction for LS can be challenging, especially for complex and lengthy strictures. For simple, first-time treated strictures, a single-stage BMGU is advocated, but many authors recognize the need for a traditional staged approach in extensively fibrotic urethras [12,13,14,15]. As a general clinical practice guide, single-stage urethroplasties do not seem to have lower success rates compared to staged urethroplasties, as described in a recent narrative review, and should therefore be performed in selected cases [16]. However, the authors state that the overall quality of the studies included is low because of the absence of a comparator, and only one non-randomized retrospective controlled study is present [17]. In this study, 1St-BMGU had a slightly significantly higher success rate than 2St-BMGU, lower operative time, complications, hospital stay, postoperative pain, and higher reported quality of life. Some more information can be extracted from extensive descriptive reports, such as the one of Andrich, Greenwell, and Mundy, where the authors evaluated the short revision rate of 1St-BMGUs and 2St-BMGUs [18]. They presented a mixed series of bulbar and penile urethroplasties, showing a higher revision rate for the 2-stage approach, which ultimately turned out to be a 3-stage urethroplasty in 50% of patients. Curiously, even if re-stricture rates were the same among 1-stage and 2-stage techniques, the authors concluded that 2-stage urethroplasty reduces recurrence. The authors justify this conclusion by reporting that in 2-stage reconstructions, 18% of patients presented with stomal stenosis after the first stage. If the procedures were a 1-stage reconstruction, it would have hesitated in a recurrent stricture, however, the 2-stage approach allowed for a revision of the first stage. Even if the number of subjects in the study was high, the mixing of bulbar and penile urethroplasties and the significant selection bias (i.e., only complex strictures underwent 2-stage reconstructions) make the interpretation of the conclusions elaborate. It is also noticeable that the authors considered 3-stage procedures as a complication rather than a stepwise approach. Another descriptive report that encompassed staged urethroplasties for LS is the one by Kulkarni, Barbagli, Kirpekar et al. [7]. The authors evaluated the results of anterior urethroplasty for LS and showed a higher treatment failure for 2-stage penile urethroplasties compared to 1-stage reconstructions. However, the number of patients in the study for these procedures was only 23, and the marked selection bias, also present in the study of Andrich, Greenwell, and Mundy, persisted; therefore, the results may be affected by the simpler strictures treated with 1-stage reconstructions. The evidence regarding single-stage compared to multistage procedures is compelling; however, it is not straightforward and generalizable. Given the low frequency of these procedures and the lack of comparison studies, it is difficult to make final statements on which surgical approach is best and on the surgical indication. It is evident, however, that in many authoritative reports, single-stage procedures lead to successful treatments. Therefore, to date, the choice between a single-stage over a multistage treatment may be committed to the surgeon based on his personal experience and, to some extent, to the patient’s preference.

This study focuses on patients for whom multistage techniques are the preferred choice in our center. To the best of our knowledge, literature regarding multistage approaches to anterior strictures is extremely scarce. We found only a few reports comparing outcomes regarding the different number of stages, and the role of the time course of the treatment was never mentioned. We show that in complex anterior strictures, where staged procedures are advocated if a first-line Johanson is performed, better treatment success is reported; also, a 3St-BMGU is more likely. The reason is that in our practice, if the first stage consisted of a Johanson only, in the second stage, we performed BMG augmentation and subsequently a third stage closure with eventual BMG revision. This stepwise approach allowed us to have an excellent evaluation of the stricture during the treatment course, providing an optimal dry healing environment and, eventually, further buccal mucosa grafting. We did not find a significant association between the number of stages and treatment outcome, likely because our sample is under numbered; nevertheless, surprisingly, a duration of the surgical management between the first and last procedure longer than 12 m seems to be a predictor of treatment success at a long-term FU. The explanation of this result may lay in the very first reason why multistage procedures were born. We suppose that if LS severely affects the urethra and surrounding tissues, the multistage approach allows a better spongiosum or graft healing under dry conditions. Likewise, if the BMGU fails, a revision can be performed in the next stage. Based on our results and experience, we agree that 1St-BMGU should be the first choice for patients without extensive involvement of the urethral plate by LS. Still, we advise that rushing the treatment course may result in worse success for complex strictures. Regardless of the number of stages adopted, surgeons should be patient and operate with the philosophy that, in complex cases, it may be better to wait more time with an opened urethral plate. It is noteworthy that three 2St-BMGUs had a treatment course longer than 12 m in our series; of these, 2 had available FU and were treated successfully. We also believe that contrary to what other authors described, the third stage in a multistage approach should not be considered a failure of the second stage but rather a natural process in the treatment of the disease. Indeed, the number and quality of studies on this topic in the scientific literature are low and well designed; prospective trials are necessary to draw final conclusions.

Although not systematically assessed, PROs are a fundamental aspect of reconstructive surgery, considering the significant impact that genital dysmorphism can have on psychological health. The only non-randomized comparative study (1St vs. 2St-BMGU) by Angulo et al. reported a better IPSS QoL domain for 1St-BMGU [17]. Erectile dysfunction (ED) is the most frequently patient-reported outcome, but it seems that neither 1St-BMGU nor staged BMGU affects this aspect [7,8,19]. In the present study, PROs do not differ between surgical approaches. We evaluated only successful treatments; otherwise, the further necessary procedures for the recurrence of stricture may have affected the answers regarding the staged procedure under study. The only expected association was found between the level of the meatus closure and the answers on the aesthetic domain. Of note, we found that sexual life quality was unchanged in 66.6% (14/19) of the patients after two or more surgeries to the penile urethra. Although remarkable, his result seems in line with the current literature on the topic [7,8,19]. A possible explanation may be that, since we used only penile urethral plate augmentation techniques, damage to the erectile physiology was unlikely. In addition, we separated aesthetic appearance from sexual life quality; therefore, any possible chordee, scarring, or retraction had felt into this domain, provided it had not strictly affected sexual life. Interestingly, the overall satisfaction with the procedure was surprisingly high (85% in the successfully treated group, Figure 2), and there was no significant difference between 2St-BMGU and 3St-BMGU. These results reinforce the consideration that patients are equally satisfied after successful treatment, even if at the cost of more prolonged discomfort.

The present study has some limitations. First, there are some missing data due to its retrospective design: FU is not available for all patients and lacks some information such as postoperative uroflowmetry results. Second, the decision toward a 2St-BMGU or 3St-BMGU may suffer from selection bias (less complicated cases may have undergone fewer stages), affecting outcome interpretation. Nevertheless, it should be noted that we reported better success for 3St-BMGU, even if not significant, and that we discussed the results concerning the duration of the treatment course rather than the type of procedure. Third, we did not use validated questionnaires for PROs assessment. Still, the use of a pre-defined questionnaire hinders possible biases thanks to the homogeneous evaluation. Fourth, our results should be interpreted in light of the small sample, which may affect the generalizability of the conclusions. This means that, in different environments, outcomes may be different; therefore, more studies on the topic are needed to add complementary evidence supporting our findings.

## 5. Conclusions

In complex cases of anterior strictures due to LS, staged approaches have satisfactory treatment success rates. Although 3St-BMGU seems to confer better outcomes, the real discriminator appears to be the duration from the first to the last stage, as more prolonged treatment courses have higher success. Surgeons should be confident in performing staged procedures and, whenever in doubt about surgical management, be aware that delaying the time of the final stage might result in better outcomes. PROs do not differ based on the number of stages performed, and overall satisfaction with the procedure is high.

## Figures and Tables

**Table 1:** Main characteristics of the population stratified by the number of stages.

		Total	2St-BMGU	3St-BMGU	*p*-Value
		*n* = 35 (100%)	*n* = 9 (25.7%)	*n* = 26 (74.3%)	
Age (months), mean (SD)		45.79 (12.80)	42.47 (14.09)	46.94 (12.40)	0.37 *
Previous urethral surgery, *n* (%)	No	4 (11%)	3 (33%)	1 (4%)	**0.044 °**
	Yes	31 (89%)	6 (67%)	25 (96%)	
Previous surgery for hypospadias, *n* (%)	No	29 (83%)	8 (89%)	21 (81%)	1 °
	Yes	6 (17%)	1 (11%)	5 (19%)	
Previous urethroplasty, *n* (%)	No	30 (86%)	9 (100%)	21 (81%)	0.30 °
	Yes	5 (14%)	0 (0%)	5 (19%)	
Previous meatoplasty, *n* (%)	No	27 (77%)	8 (89%)	19 (73%)	0.65 °
	Yes	8 (23%)	1 (11%)	7 (27%)	
Previous dilatations, *n* (%)	No	15 (43%)	4 (44%)	11 (42%)	1 °
	Yes	20 (57%)	5 (56%)	15 (58%)	
Previous urethrotomy, *n* (%)	No	27 (77%)	6 (67%)	21 (81%)	0.40 °
	Yes	8 (23%)	3 (33%)	5 (19%)	
Qmax before surgery (mL/s), median (IQR)		6 (4.6–7)	6 (4–6.5)	5 (4.6–7)	0.82 *
First-stage surgery, *n* (%)	Johanson only	28 (77%)	3 (33%)	25 (96.1%)	<0.001 °
	Other	7 (23%)	6 (67%)	1 (3.9%)	
Months between first and last stage, *n* (%)	<12 m	8 (23%)	6 (67%)	2 (8%)	0.001 °
	>12 m	27 (77%)	3 (33%)	24 (92%)	

The table summarizes the main characteristics of all the population that underwent a St-BMGU stratified by the number of stages. Percentages refer to the column. Bold *p*-values are significant. * = Mann–Whitney test. ° = Fisher’s exact test.

**Table 2:** Main characteristics of the population with available follow-up.

		Total	Treatment Failure	Treatment Success	*p*-Value
		*n* = 25 (100%)	*n* = 5 (20%)	*n* = 20 (80%)	
Age (years), mean (SD)		46.02 (11.12)	42.25 (19.02)	46.96 (8.68)	0.41 *
Previous urethral surgery, *n* (%)	No	4 (16%)	2 (50%)	2 (50%)	0.17 °
	Yes	21 (84%)	3 (14.3%)	18 (85.7%)	
Previous surgery for hypospadias, *n* (%)	No	23 (92%)	4 (17.4%)	19 (82.6%)	0.37 °
	Yes	2 (8%)	1 (50%)	1 (50%)	
Previous urethroplasty, *n* (%)	No	21 (84%)	4 (19%)	17 (81%)	1 °
	Yes	4 (16%)	1 (25%)	3 (75%)	
Previous meatoplasty, *n* (%)	No	19 (76%)	4 (21.1%)	15 (78.9%)	1 °
	Yes	6 (24%)	1 (16.7%)	5 (83.3%)	
Previous dilatations, *n* (%)	No	12 (48%)	3 (25%)	9 (75%)	0.64 °
	Yes	13 (52%)	2 (15.4%)	11 (84.6%)	
Previous urethrotomy, *n* (%)	No	20 (80%)	4 (20%)	16 (80%)	1 °
	Yes	5 (20%)	1 (20%)	4 (80%)	
Qmax before surgery (mL/s), median (IQR)		6 (4.8–8.5)	6 (6–6)	6 (4.6–10)	0.88 *
Number of stages, *n* (%)	2St-BMGU	6 (24%)	3 (50%)	3 (50%)	0.07 °
	3St-BMGU	19 (76%)	2 (10.5%)	17 (89.5%)	
First-stage surgery, *n* (%)	Johanson only	21 (84%)	2 (9.5%)	19 (90.5%)	<0.001
	Other	4 (16%)	3 (75%)	1 (25%)	
Months between first and last stage, *n* (%)	<12 m	6 (24%)	4 (66.7%)	2 (33.3%)	0.005 °
	>12 m	19 (76%)	1 (5.3%)	18 (94.7%)	

The table summarizes the main characteristics of the population that underwent a St-BMGU with available FU stratified by treatment outcome. Percentages refer to the row except for the percentage of the “Total” column, which refers to the column. * = Mann–Whitney test. ° = Fisher’s exact test.

**Table 3:** Univariable and multivariable logistic regression models, predictors of treatment failure.

**Univariable model**	**Coef.**	***p*-Value**	**95% CI**	
Treatment course (<12 m vs. >12 m)	36	0.008	2.585	501.268
Number of stages (2St-BMGU vs. 3St-BMGU)	8.5	0.053	00.971	74.424
**Multivariable model**	**Coef.**	***p*-value**	**95% CI**	
Treatment course (<12 m vs. >12 m)	27	0.031	1.359	537.553
Number of stages (2St-BMGU vs. 3St-BMGU)	1.743	0.715	0.089	34.231

The table shows the predictors of treatment failure in logistic regression models.

## Data Availability

Data are available under request to the corresponding author.
